# Modulation of electronic transport properties in armchair phosphorene nanoribbons by doping and edge passivation

**DOI:** 10.1038/s41598-017-13212-7

**Published:** 2017-10-09

**Authors:** Caixia Guo, Tianxing Wang, Congxin Xia, Yufang Liu

**Affiliations:** 1 College of Physics and Materials Science, Henan Normal University, Xinxiang, Henan, 453007 China; 2 College of Electronic and Electrical Engineering, Henan Normal University, Xinxiang, Henan, 453007 China

## Abstract

The electronic structures and transport properties of group IV atoms (C, Si, Ge)-doped armchair phosphorene nanoribbons (APNRs) are investigated using first-principles calculations, considering different edge passivation. The results show that the C, Si, Ge dopants can induce the transition occur from semiconductor to metal in the APNRs. The negative differential resistance (NDR) behavior in the doped APNR system is robust with respect to the doping concentration and edge passivation type. However, their current peak positions and peak-to-valley ratio (PVR) values are correlated with doping concentration and edge passivation type. In particular, for the C, Si-doped APNRs, the low bias NDR behavior with the PVR (10^5^–10^8^) can be observed when doping concentration is low in the APNRs with the F and H edge passivation. These results may play an important role for the fabrication of future low power consumption nano-electronic devices.

## Introduction

Since the graphene was firstly fabricated by mechanical exfoliation^1^, many interesting electronic properties of the atomically thin two-dimensional (2D) materials have attracted considerable attentions^2–15^. Among these electronic properties, negative differential resistance (NDR) behavior has wide range of applications in electronic devices, including frequency multiplier, electronic switches, oscillator, and so on^16–18^. NDR behaviors of 2D materials-based electronic devices have been studied in both experiments and theoretical fields. However, most of current peak-to-valley ratios are not excess of the order of 10^2^, which limits their practical applications in the electronic devices and stimulates us to search new 2D materials^19–25^.

Recently, few-layer black phosphorus also was successfully fabricated, and becomes another kind of promising 2D semiconducting material^26^. The few-layer black phosphorus has a tunable direct band gap and a strong in-plane anisotropic nature, which endow it with inimitable glamour^27,28^. Due to the semiconducting feature, phosphorene may be superior to graphene for electronic device applications in some ways. For example, few-layer phosphorene based field effect transistors have higher mobility and a high on/off ratio^29^. Owing to these unique physical properties, the study of phosphorene and phosphorene nanoribbon (PNR) attracts a great of research interest for various device applications^30–38^. Li *et al*. demonstrated that hydrogenated PNR were promising candidates for applications in electronic and optoelectronic devices^35^. Zhang *et al*. revealed nonlinear current-voltage behaviors and the NDR in a homostructured device based on zigzag PNR^39^, but the current peak-to-valley ratios are also not excess of the order of 10^2^. In addition, our previous works found that the NDR behaviors can be observed in the C, Si, Ge-doped phosphorene monolayer with obvious anisotropic^40^. Peng *et al*. studied the edge and quantum confinement effects on the electronic properties of the PNR, and they found that APNRs are semiconductors for all edge groups^41^.

Nonetheless, it is rare to find studies on modulation of the transport properties in the APNR-based devices. As mentioned above, the NDR behavior is very important for the nano-electronic devices. Thus, if one can find a way to effectively modulate the NDR behavior of the APNR, it will bring out interesting application potentials for this material. In this work, we employ first-principles calculations and non-equilibrium Green’s function to study the transport properties of the edge hydrogen passivated APNRs containing C, Si, Ge impurities. The numerical results show that robust NDR behavior always can be observed in these doped APNR based devices, and the doping concentration can strongly affect the NDR behavior of the doped APNRs devices. Then, the transport properties of the C-doped APNRs are investigated by tuning the edge passivation type. Interestingly, the edge passivation type can future modulate the NDR behaviors in the C-doped APNR-based devices.

## Results

### The electronic structures of doped APNRs

**Figure 1:**
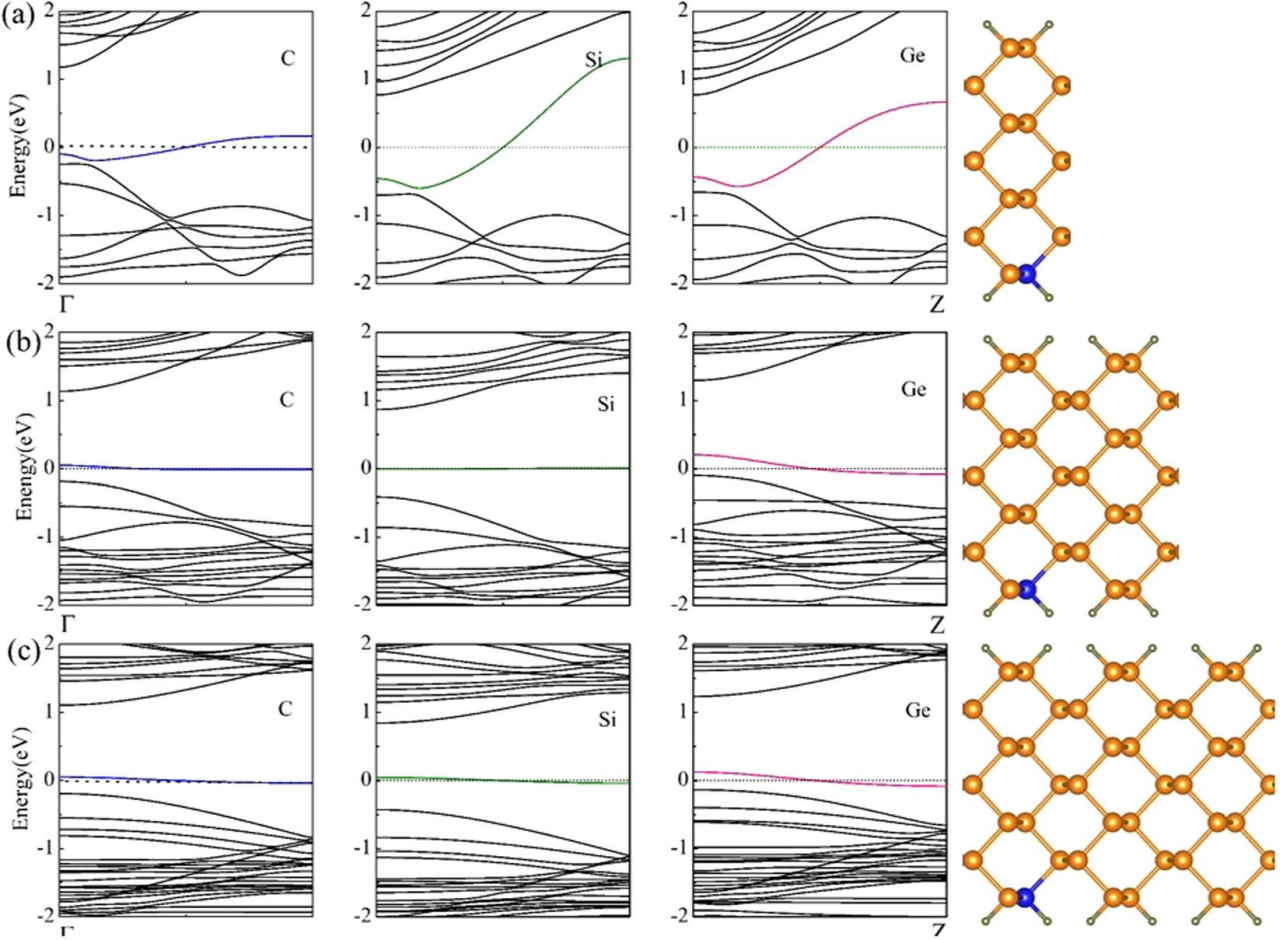
Calculated band structures of doped APNRs with different doping concentrations of (**a**) 7.1%, (**b**) 3.6% and (**c**) 2.4%, respectively. The right panels in each figure show the schematic doped APNRs, respectively. The horizontal zero line represents the Fermi level.

Based on the previous report^42^, the formation energies of substitutional dopants in phosphorene show that the C, Si, Ge substituting P can be thermodynamically stable in the relevant systems. Thus, in the following, we only consider the case of C, Si, Ge substituting P. In order to find out the modification of band structures affected by substitutional doping, Fig. 1(a)–(c) presents the band structures of the group IV atoms-doped 7-APNRs with all edge P atoms terminated by H atoms, where 7 is the number of P atoms across the ribbon width. According to previous calculations, APNRs with different width are semiconductors, and electronic band gaps increases monotonously with decreasing the ribbon width. We choose the 7-APNR as object, due to it has a wider direct band gap. In Fig. 1(a), after the one edge P atom per one unit cell is substituted by C atom, which corresponds to doping concentration of 7.1%, the band structure of the C-doped APNR show metallic behavior because a half-filled impurity-band crossing the Fermi level was observed. In addition, the band structure of APNR with the Si/Ge atom displaces an edge P atom is also illustrated in the Fig. 1(a). Interestingly, the bandwidth of the impurity-band crossing the Fermi level becomes larger in the Si/Ge-doped APNR. When one edge P atom per two unit cells is substituted by C, Si, Ge atoms in APNRs, which corresponds the doping concentration of 3.6%, the band structures of these APNRs are shown in Fig. 1(b). We can see that these bandwidths of impurity-bands crossing the Fermi level decrease with decreasing the doping concentration. The similar phenomenon is also can be found in the band structures of doped APNRs with one edge P atom per three cell units is substituted by impurity atom, as shown in Fig. 1(c). These results may imply that the bandwidth of the impurity-band vary with the doping concentration in APNRs, and the C, Si, Ge atoms can induce the transition occurs from semiconductor to metal in the APNRs, irrespective of doping concentration.

**Figure 2:**
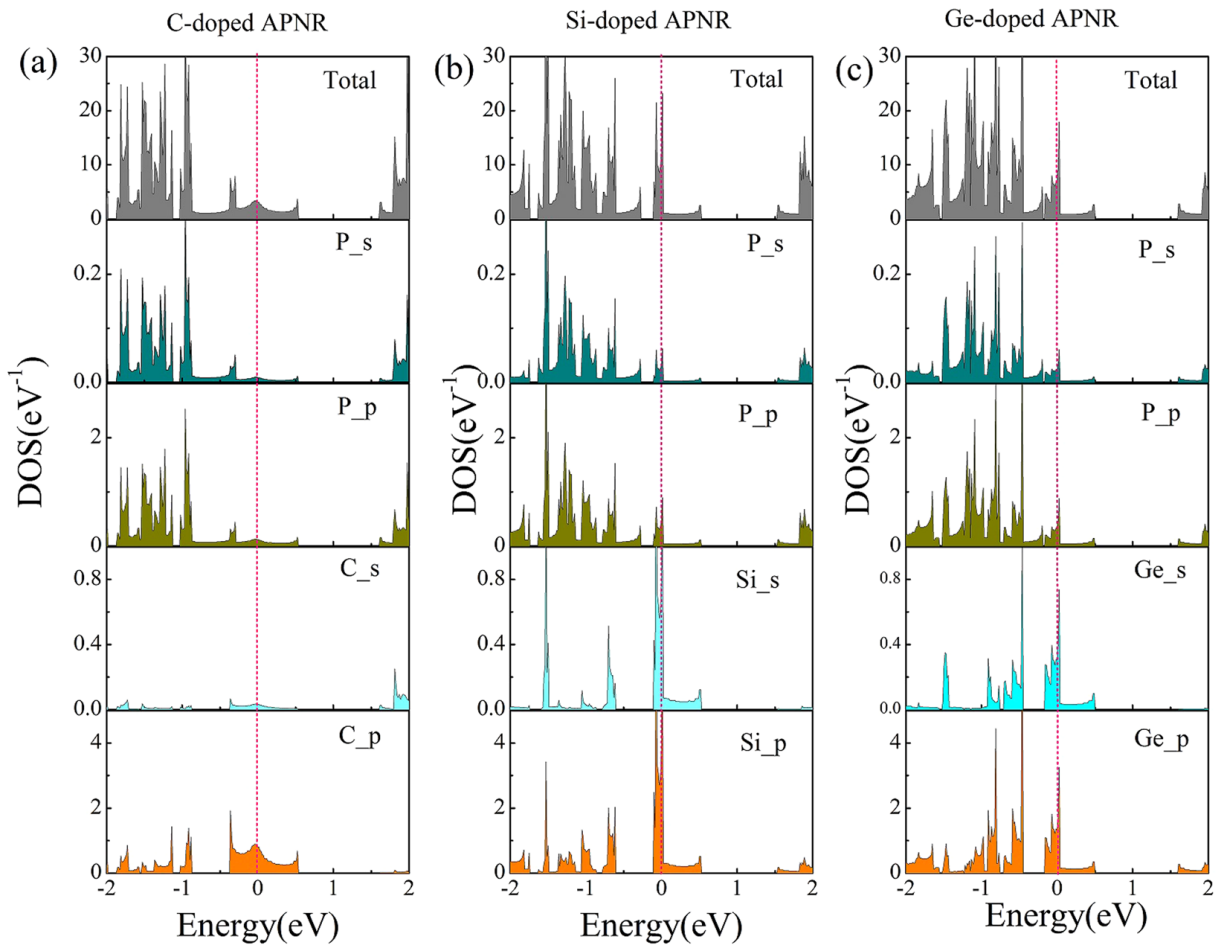
The total DOS and partial DOS of doped APNRs with (**a**) C atom, (**b**) Si atom and (**c**) Ge atom, respectively. The partial DOS projected onto the s and p orbitals of the dopant and P atom are displayed. The energy zero represents the Femi level.

Take the APNRs with one center P atom per unit cell is substituted by one dopant atom for example, Fig. 2(a)–(c) show the total DOS and partial density of states (PDOS) projected onto the s and p orbitals of P atom and each dopant atom. The similar pattern and peak positions between the PDOS of the dopants and P atoms, indicating a strong hybridization of *sp* orbitals between dopant atoms and P atoms. The partial DOS peaks of the C, Si, Ge dopants and P atoms locate at the Fermi level, thereby giving rise to a metallic property. This phenomenon further demonstrate that the metallic behavior of the doped APNRs originates from the impurities atoms.

### The transport characteristics of APNR based devices

**Figure 3:**
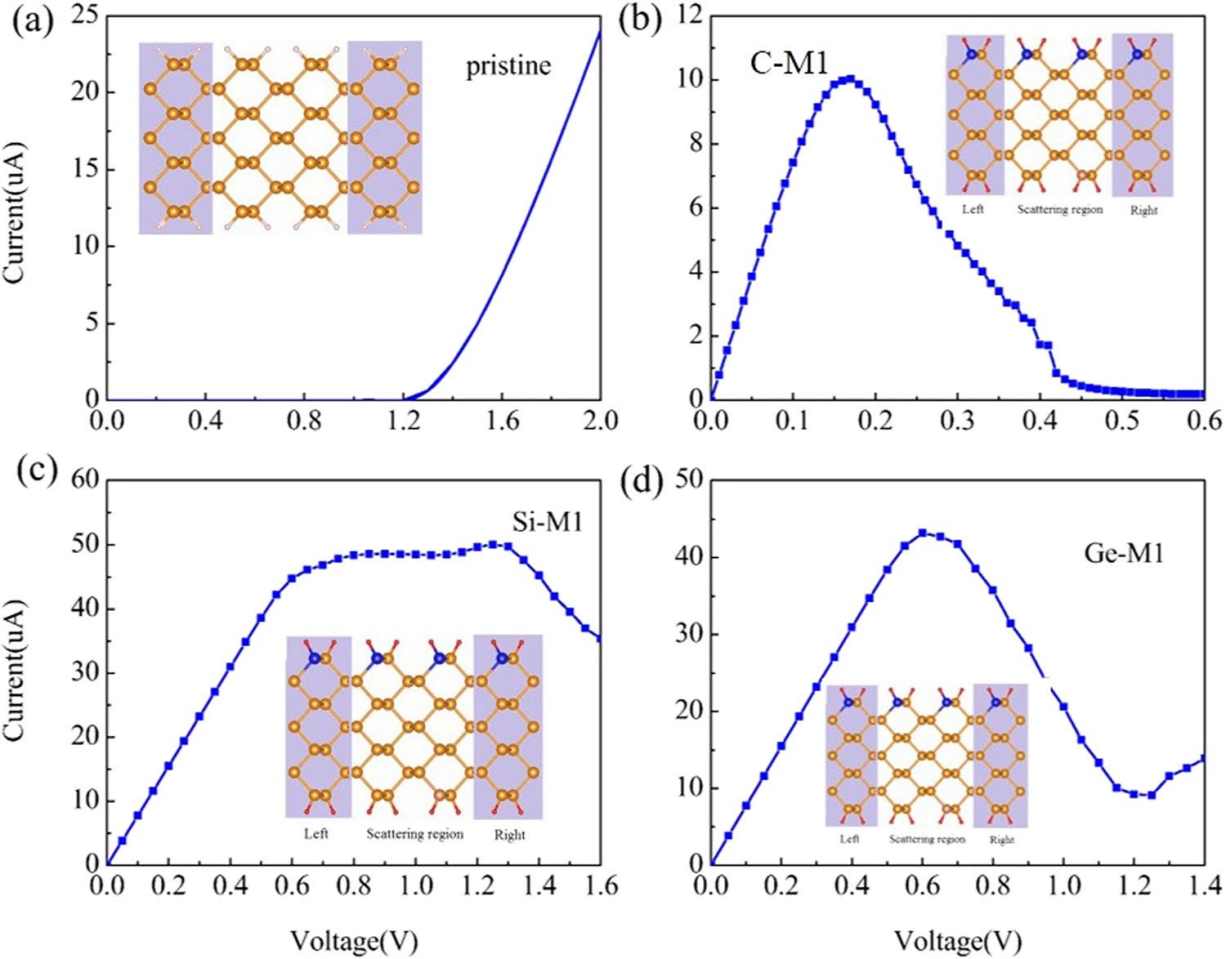
Calculated I-V curves of (**a**) pristine and (**b**)–(**d**) C, Si, Ge-doped APNR based device M1, respectively. The orange, grey, and blue balls represent P, H, and dopants atoms, respectively.

According to theoretical previous studies of the transport properties of doped-nanoribbons^19,20,43–45^, such as C-doped armchair boron nitride nanoribbons and B, N-doped graphene nanoribbons. We construct the two-probe models of pristine APNR and doped APNR with C, Si, Ge atoms, named M1, are illustrated in Fig. 3(a)–(d). The left and right electrodes are indicated by the shadowed areas, between which is the center scattering region. We can see that the model structure M1 consists of three distinct regions: two semi-infinite electrodes and a central scattering region, and the scattering region is the expansion of the electrodes by directly connecting. To investigate the influences of dopants on the transport properties of the APNR-based device, Fig. 3(a,b) present the characteristics of the current-voltage (I-V) of M1. For the pristine APNR-based device, Fig. 3(a) shows that current values are always zero until the bias voltage reach to 1.2 V, which is in excellent agreement with the direct band gap value of 1.2 eV. For the C-doped device M1, we can see the current increase quickly to the value maximum at the bias voltage 0.17 V, and then decrease with the bias and reduce to value minimum at the bias 0.6 V, thus a NDR behavior can be observed and the corresponding current peak-to-valley ratio (PVR) is 52, as shown in Fig. 3(b). However, for the Si-doped APNR-based device M1, we can see that the current increase monotonously to the value maximum at the bias 1.25 V, and then decrease to value minimum at the 1.9 V, the current PVR only reaches to 2, as shown in Fig. 3(c). For the Ge-doped APNR-based device, the obvious NDR behavior also can be observed. The current peak locates at the 0.6 V and the current PVR is 5. Through analysis the I-V curves of the doped APNR based devices, we can found that NDR behavior always can be found in C, Si, Ge-doped APNR-based devices. However, the observed current PVR values of these devices are all not excess the order of 10^2^, which limits the use of group IV atoms-doped APNRs in electronic devices.

## Discussion

**Figure 4:**
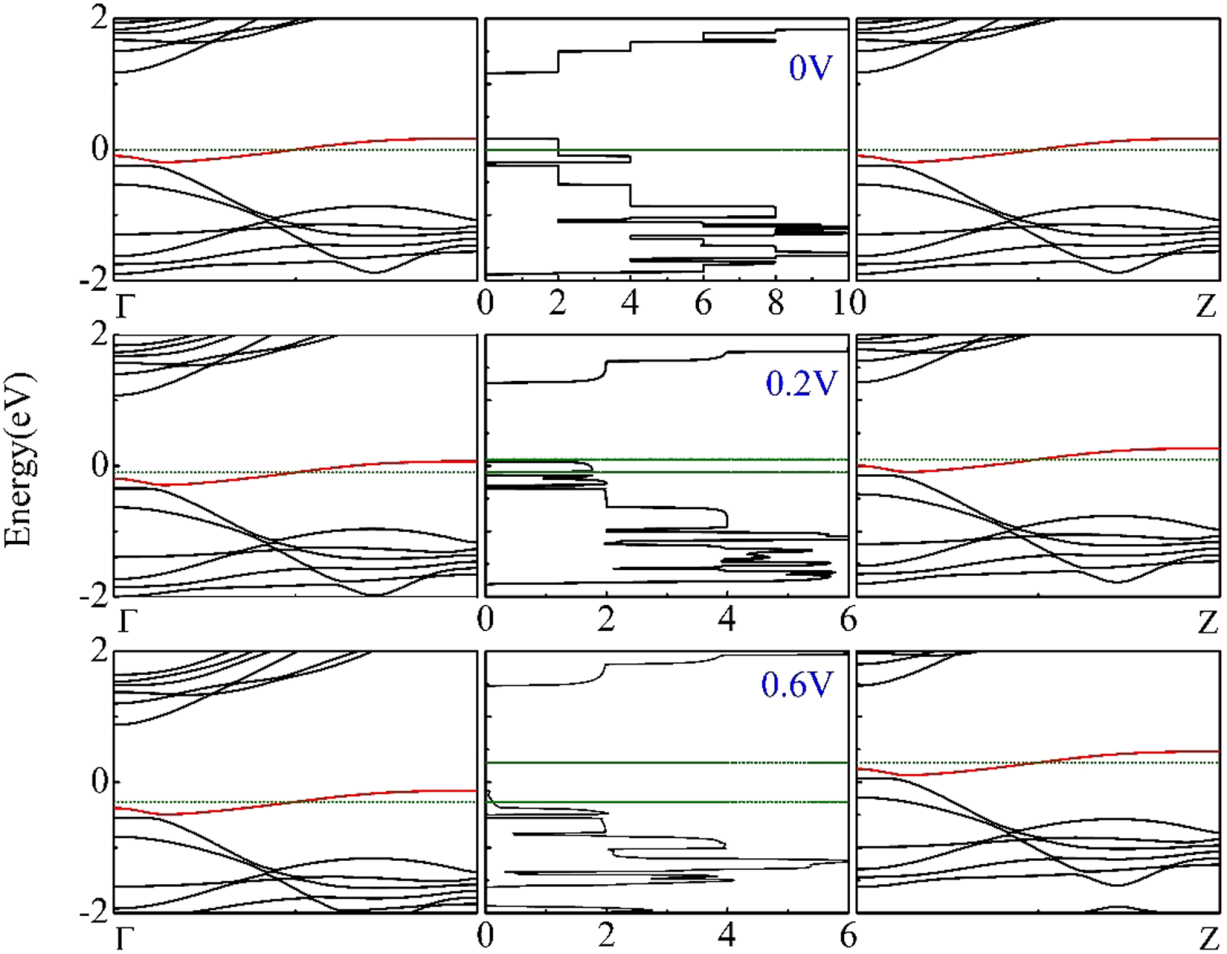
Transmission spectra and the band structures of both electrodes at three difference bias 0 V, 0.2 V, 0.6 V for the edge C-doped APNR device M12. The horizontal olive lines represent the electrochemical potentials of the left and right electrodes. The Fermi level is set to be zero.

In order to investigate the origination of the obvious NDR behavior, we take the C-doped APNR-based device (C-M1) as an example, Fig. 4 presents the bias-dependent transmission spectra and the band structures of both electrodes at the bias 0 V, 0.2 V, 0.6 V, respectively. Here, we can see clearly that a C atom-induced impurity-band (denote as red line) crossing the Fermi level, forming a so called metallic state, which will attribute to the conductance of this device. Here we would like to point out that C atom-induced metallic state are different from the normal metallic state. The reason can be given as follows. Firstly, this impurity-band is isolated. There is no overlap with other bands from the host material of black phosphorus. It is fully coming from the ‘carbon doping’ in this model, which shows strong quantum confinement effect. Secondly, this impurity-band is very narrow (it’s width is about 0.2 eV) and rather flat, which is a character of a weak interaction system with poor conductivity. Also, these characters can be further proved by the bias dependent transmission spectra as shown in Fig. 4. We can clearly see the integral steps of the transmission with different energy, showing the conductance of different channels corresponding to the different energy levels in the system. These channels can open or close depending on the bias window and level match between the two “fake electrodes” here. Under zero bias, the level match is perfect, thus the high transmission is found. But no current from the zero bias. With bias increased to 0.2 V, more opened conductance channels in the large bias window give high current, as shown in the Fig. 3(b). When bias further increase above to 0.4 V, the band match will no longer happen, though the bias window become larger. Thus, the very weak transmission and low current is found. Since the electrons cannot jump to other bands in the bias window from the unmatched symmetry. When the bias reaches to the 0.6 V, the current values of C-doped device decrease to nearly zero which contribute to the giant PVR of NDR. Therefore, before the bias reaches to the 0.2 V, the current increase rapidly from the band match and increased number of open channels. After that, the current decreases quickly from the band mismatch. Therefore, the NDR behavior occurs, which is usually the result of asymmetrical transmission on energy as shown here.

### Doping concentration tuning of NDR behavior in doped APNRs

**Figure 5:**
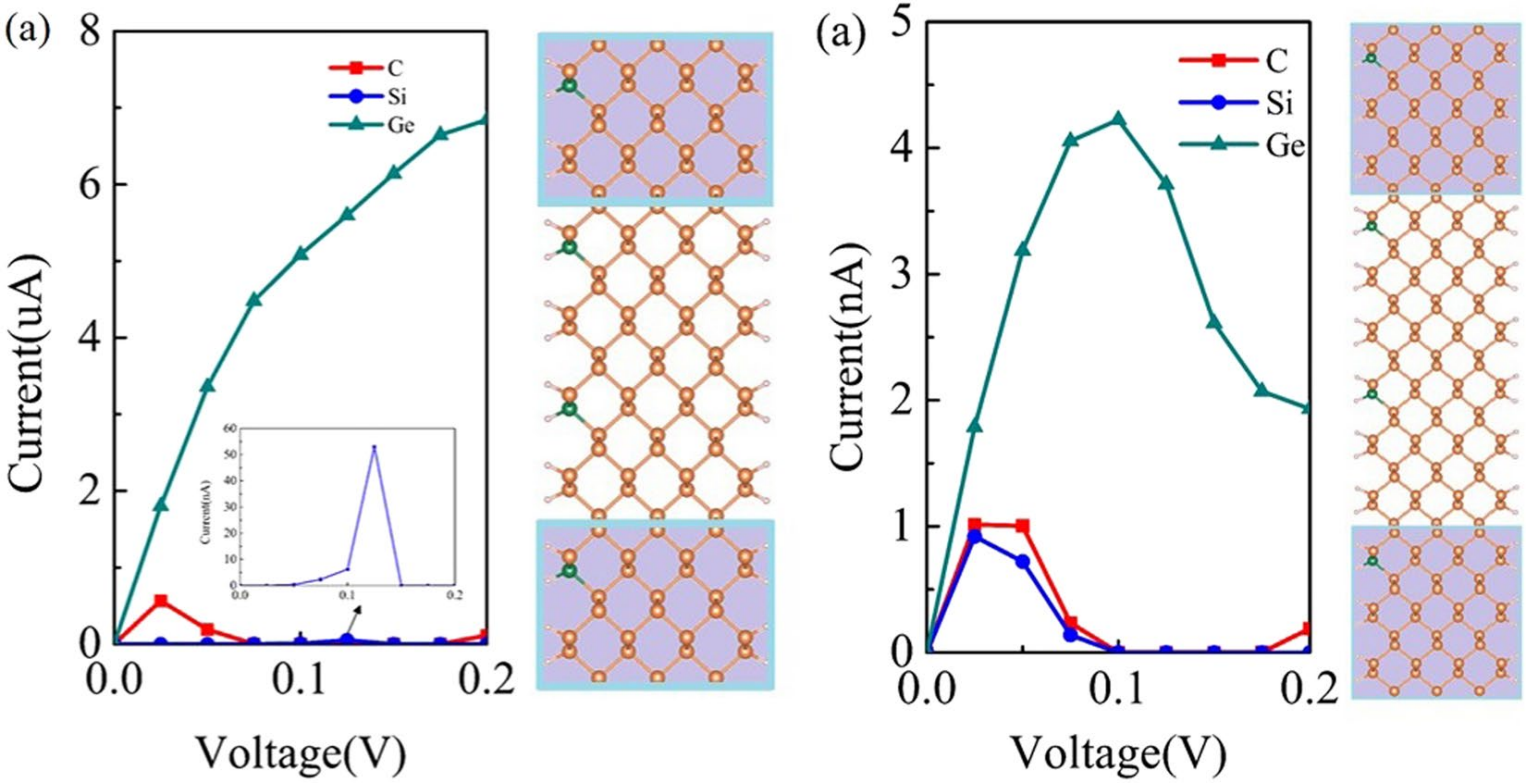
Calculated I-V curves of C, Si, Ge -doped APNR devices M2 (**a**) and M3 (**b**). The right panels in each figure show the schematic doped APNR devicesM2 and M3, respectively.

To explore the effects of the doping concentration on the transport properties of these doped APNRs, we calculated the I-V curves of M2 and M3 with a small doping concentration of 3.6% and 2.4%, as shown in Fig. 5(a,b). For the M2, which one edge P atom per two unit cells is substituted by one dopant atom, NDR behaviors can be observed in Fig. 5(a). The numerical result show that the current values of the Ge-doped device is biggest in the bias region [0 V, 0.2 V] among the three devices. While, it’s current PVR only reaches to 3. For the Si-doped device M2, the current peak value locates at the 0.125 V and the PVR reaches to 7, which is not large enough for application in nano-electronic device. For the C-doped device M2, the current peak value locates at the 0.025 V and the PVR reaches to the order of 10^2^. Compared with Si, Ge-doped device M2, the C-doped device M2 possess the lowest bias NDR and the largest current peak-to-valley ratio.

Continue to decrease the doping concentration, In Fig. 5(b), for C, Si, Ge-doped devices M3 with one edge P atom per three unit cells is substituted by one dopant atom, it is easy to find that obvious NDR behaviors occur in these devices, and the PVR values of them reach to order of 10^4^,10^8^ and 10^1^, respectively. In particular, for the Si-doped device M3, the PVR value reaches to the order of 10^8^, which are much larger than those reported values in doped graphene nanoribbons^22^. Compared with devices M1, M2 and M3, the current peak positions of these devices move down toward the lower bias values with decreasing the doping concentration, i.e. the current peak positions of C-doped devices M1 and M2 locate at 0. 17 V and 0.125 V, while 0.04 V is peak position of C-doped device M3. Moreover, the current PVR quickly increases with decreasing the doping concentration. i.e. the current PVR values of C-doped devices M1 and M2 are not excess the order of 10^3^, while the PVR of M3 reaches to the order of 10^4^. Thus, we can conclude that robust NDR behaviors of group IV atoms-doped APNRs do not depend on doping concentration. However, the current peak position move down toward the lower bias with decreasing the doping concentration in the C, Si-doped APNR-based devices. These numerical results may imply that C, Si-doped APNRs are promising candidates for the future application in the low power consumption electronic device.

### Edge passivation type tuning of NDR behavior in C-doped APNRs

Despite the giant PVR in the low bias can be observed in the hydrogen passivated M3 with a small C, Si doping concentration of 2.4%, we hope to further improve the NDR effect in the C-doped APNR-based device. Motivated by that the edge types of the ribbons play a critical role on their electronic properties, we first studied the stability of the fluorine and chlorine passivated APNRs. For comparison, the hydrogen passivated APNR also is shown here. The stability of these APNRs are studied by the formation energies ${E}_{form}$, which is defined as:

$${E}_{form}\,=\,{E}_{tot}\,-\,{E}_{bare}\,-\,{n}_{X}{E}_{{X}_{2}}/2,$$

where the ${E}_{tot}$ and ${E}_{bare}$ are the total energies of APNRs with edge passivation and bare edge, respectively. ${E}_{{X}_{2}}$ is the total energy of an isolated X2 (X = H, F, Cl) molecule. ${n}_{X}$ is the number of X atoms. The formation energies of hydrogen, fluorine and chlorine passivated APNRs are −3.8 eV, −3.5 eV and −1.75 eV per unit, respectively. The negative formation energies indicate that APNRs with H, F, Cl edge passivation are all energetically stable.

**Figure 6:**
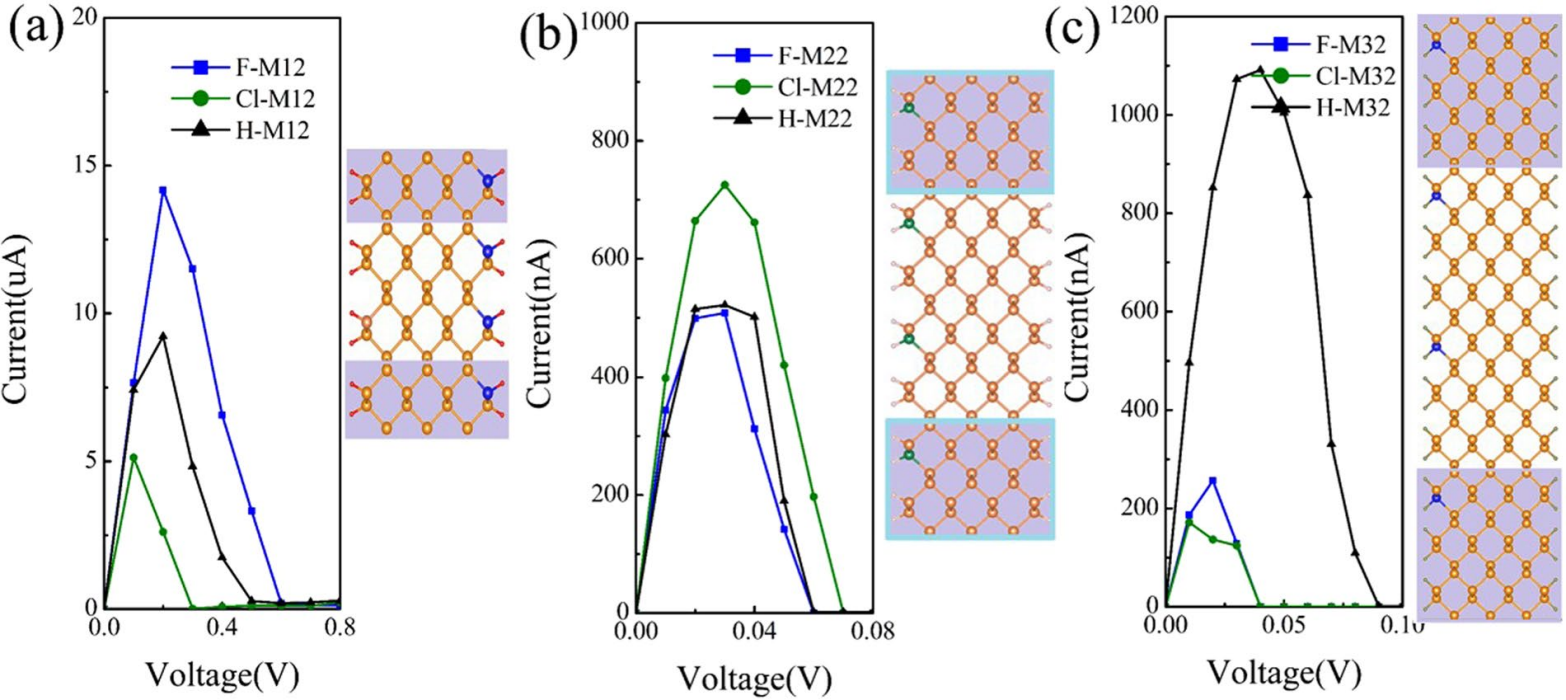
Calculated I-V curves of C-doped APNR devices with different edge passivation types. (**a**) The edge hydrogen, fluorine and chlorine passivated M1, respectively. (**b**) The edge hydrogen, fluorine and chlorine passivated M2. (**c**) The edge hydrogen, fluorine and chlorine passivated M3. The right panels in each figure show the schematic M1, M2 and M3, respectively.

Then, the I-V characteristics of the F and Cl passivated APNR-based devices containing C impurities are investigated. Figure 6(a) presents the I-V curves of the M1 with the edge passivated by F and Cl atoms, respectively. For comparison, the I-V curves of the M1 with edge H passivation also is illustrated in the Fig. 6(a). It is clear to see that obvious NDR behaviors also can be found in these devices. The current peak positions of the F, H and Cl passivated systems locate at the 0.17 V, 0.17 V and 0.12 V, respectively, and the relevant PVR values of these devices are 115, 52 and 1617, correspondingly. As mentioned above, the doping concentration can affect the NDR behavior in the doped APNR-based device. Therefore, the I-V characteristics of the F and Cl passivated APNR-based device M2 are also investigated in order to further modulate the lower bias NDR effect. In Fig. 6(b), for the F and Cl passivated M2, the current peak positions locate at the 0.03 V and 0.03 V, and the PVR values of NDR behaviors up to the order of 10^6^ and 10^4^, respectively. Figure 6(c) presents the I-V curves of the H, F and Cl passivated M3. The current peak positions locate at the 0.04 V, 0.02 V and 0.01 V, and the relevant PVR of the NDR behaviors up to 10^4^,10^4^ and 10^5^, correspondingly. These numerical results indicate that the edge Cl passivation can further induce giant NDR behavior in the lower bias.

## Discussion

As mentioned above, we know that the matching and/or mismatching of impurity-bands between two electrodes induce the NDR behavior occurs, and the peak positions would locate at lower bias when the impurity-band’s widths of the electrodes are narrower. The calculated impurity-band’s widths of electrodes in the C-doped devices with different edge passivation types, are shown in Table 1. It can be found that the bandwidths of impurity-band gradually decrease in the Cl passivated APNRs with decreasing the doping concentration. The similar phenomenon is also observed in the H passivated APNRs. However, when the doping concentration is same, the bandwidths of impurity-bands in Cl passivated APNRs are narrower than that in H passivated APNRs, which implying that the NDR behaviors in the Cl passivated APNRs can be realized at relative low bias region. Therefore, with the decrease of doping concentration, we can infer that the lower bias NDR behavior should be formed in the Cl passivated APNR devices. This result may imply that the C-doped APNR with edge Cl passivation is proper to fabricate the lower power consumption electronic devices.

**Table 1:** Impurity band’s widths of C-doped APNRs with the edge passivated by H and Cl, respectively, considering different doping concentration.

Doping concentration (%)	7.1	3.6	2.4	1.2	1.0	0.9	0.8	0.7
Bandwidths of Cl-APNR (meV)	170	71	36	3.2	0.91	0.61	0.38	0.18
Bandwidths of H-APNR (meV)	360	90	60	19	12	8	6	4

Finally, we would like to point out that there are many theoretical studies on the NDR behavior in PNRs^39,40,44^, but the relative experimental studies are still missing. Recently, shim *et al*. demonstrated a NDR dffects in the phosphorene/rhenium disulfide heterojunction^46^. We expect that with the development of experimental technologies, the NDR effects can be realized in nanoribbon-based electronic devices in near future.

## Conclusions

In summarily, we have studied the modulation of transport properties of group IV atoms-doped APNRs. The robust NDR behaviors in the C, Si, Ge-doped APNRs can be modulated by tuning the doping concentration and edge passivation type. Interestingly, compared with H and F passivated APNRs, the Cl passivated APNRs have the giant PVR in the lowest bias voltage region. We expect that our calculations can be helpful to related physical and device studies, and support the possibility of potential applications in low power consumption nanoscale devices.

## Method

In this work, geometry relaxations, electronic structures and transport properties are calculated using self-consistent density functional theory as implemented in the Atomistix ToolKit (ATK) package^47,48^. The Perdew-Burke-Ernzerhof (PBE) formulation of the generalized gradient approximation (GGA) is used as the exchange-correlation functional. The energy cutoff for the plane waves is 150 Ry and the Brillouin zone was sampled using a $1\times 1\times 50$ Monkhorst-pack grid. The geometry relaxations of above systems are performed until the absolute value of force acting on each atom is less than 0.01 eV/Å. Periodic boundary conditions and vacuum spaces of at least 15 Å are imposed on the plane perpendicular to the z axis of the APNR. A k-points grid of $1\times 1\times 100$ in the x, y, and z directions is adopted during the transport calculations. The electrons temperature used is 300 K. The current through the device is calculated using the Landauer-Büttiker formula^49^:

$$I(V)\,=\,\frac{2e}{h}{\int }_{-\infty }^{+\infty }T(E,V)[{f}_{L}(E,{\mu }_{L})-{f}_{R}(E,{\mu }_{R})]dE,$$

where $E$ is the energy of the electron, ${f}_{L(R)}(E,{\mu }_{L})$ is the equilibrium Fermi distribution for the left (right) electrode, ${\mu }_{L,R}\,=\,{E}_{F}\pm \frac{eV}{2}$ are the electrochemical potentials of the left and right electrodes in terms of the common Fermi energy ${E}_{F}$. The $h,V$ and $T(E,V)$ is Planck constant, bias and transmission coefficients, respectively.
